# Supplementary material for: Patterns of Intron Gain and Loss in Fungi
Source: PLoS Biol. 2004 Nov 30;2(12):e422. doi: 10.1371/journal.pbio.0020422 (PMC532390; doi:10.1371/journal.pbio.0020422)
Supplement: Table S1 — Also available at http://genes.mit.edu/NielsenEtAl/. (4.3 MB ZIP). [file pbio.0020422.st001.zip › NielsenEtAl/html/677.html]

AN4762.1.NCU02016.1.MG03663.1.FG08530.1


```
 CLUSTAL W (1.82) Multiple Sequence Alignments - Introns Inserted


Sequence 1: NCU02016.1	128 aa
Sequence 2: MG03663.1	129 aa
Sequence 3: FG08530.1	128 aa
Sequence 4: AN4762.1	144 aa
Alignment Length: 144 aa
Number Identitical Residues: 51 aa
Alignment Score (without introns) 2713


MG03663.1 	MAS---FIARRAFT------TSARR-LEAGAKEHVLKQEGKKDPELM0ILGGIMVAAFAG
NCU02016.1	MAS---FIARRAFS------TTVRR-MTTG--EEALKTESKKNPEIL0VLGGVMVAAFIG
FG08530.1 	MASR--IVSRRFLS------TTARR-FQEHQ-KSELKKESQRNPEIL0ILGGVMVAALGG
AN4762.1  	MSSIAAMASRRAFARQALFRAPVRRAYSSKMEESTLDKAPKRDPELY0VLLGVMSGAFLI
          	*:* ::: :** :: .:   :..**:     .:  *.   :::**:  :* *:* .*:  

MG03663.1 	AGF~YFG1RTPTSSTSETKVPMAKDAMPWESET---AGKYKYHPGGDPNAAPKDAPSAVN
NCU02016.1	AGY~YFA1RNPTASTAETAVPQTKNSMPWESGSG--AGKYQYHPGGDPSAAPKDAPSAVN
FG08530.1 	AGY~YLG1RSPTGATSESPVTIAT--HPWESGS---SGKYQYHPGGDLSQAPKDAPSALN
AN4762.1  	AGW2YFG~KKPTSVTSESNVRIPDSSMPWERDDDGKIYKYQYHPGGDKSQPLRNAPSALN
          	**: *:. :.**. *:*: *  . .: ***   ...  **:****** . . ::****:*

MG03663.1 	VVVVPDVTLPK0NLHDKWNKWGKEGY--
NCU02016.1	VVVVPNVTLPK0SLHEKYNKWGKEGY--
FG08530.1 	VVVVPNVTLPS~ELHDKYNKWGKDGYP-
AN4762.1  	TVIVPNVTLPV0DLHERFNKYGKEEYDF
          	.*:**:****  .**:::**:**: *
```
